# Early Detection of Junctional Adhesion Molecule-1 (JAM-1) in the Circulation after Experimental and Clinical Polytrauma

**DOI:** 10.1155/2015/463950

**Published:** 2015-10-18

**Authors:** Stephanie Denk, Rebecca Wiegner, Felix M. Hönes, David A. C. Messerer, Peter Radermacher, Manfred Weiss, Miriam Kalbitz, Christian Ehrnthaller, Sonja Braumüller, Oscar McCook, Florian Gebhard, Sebastian Weckbach, Markus Huber-Lang

**Affiliations:** ^1^Department of Orthopedic Trauma, Hand, Plastic and Reconstructive Surgery, University Hospital of Ulm, 89081 Ulm, Germany; ^2^Institute of Pathophysiology and Process Development in Anesthesia, University of Ulm, 89081 Ulm, Germany; ^3^Department of Anesthesiology, University Hospital of Ulm, 89081 Ulm, Germany; ^4^Department of Orthopedic Surgery, University of Ulm, RKU, 89081 Ulm, Germany

## Abstract

Severe tissue trauma-induced systemic inflammation is often accompanied by evident or occult blood-organ barrier dysfunctions, frequently leading to multiple organ dysfunction. However, it is unknown whether specific barrier molecules are shed into the circulation early after trauma as potential indicators of an initial barrier dysfunction. The release of the barrier molecule junctional adhesion molecule-1 (JAM-1) was investigated in plasma of C57BL/6 mice 2 h after experimental mono- and polytrauma as well as in polytrauma patients (ISS ≥ 18) during a 10-day period. Correlation analyses were performed to indicate a linkage between JAM-1 plasma concentrations and organ failure. JAM-1 was systemically detected after experimental trauma in mice with blunt chest trauma as a driving force. Accordingly, JAM-1 was reduced in lung tissue after pulmonary contusion and JAM-1 plasma levels significantly correlated with increased protein levels in the bronchoalveolar lavage as a sign for alveolocapillary barrier dysfunction. Furthermore, JAM-1 was markedly released into the plasma of polytrauma patients as early as 4 h after the trauma insult and significantly correlated with severity of disease and organ dysfunction (APACHE II and SOFA score). The data support an early injury- and time-dependent appearance of the barrier molecule JAM-1 in the circulation indicative of a commencing trauma-induced barrier dysfunction.

## 1. Introduction

Polytrauma with subsequent complications, such as systemic inflammation and multiple organ dysfunction and failure, remains a leading cause of mortality among people aged 45 and younger [1]. Immediately after the trauma impact, the injured patient is exposed to multiple danger- and pathogen-associated molecular patterns, leading to molecular, cellular, and organ dysfunction [2]. A main pathophysiological driver for multiorgan failure is the dysfunction and breakdown of external (skin) and internal barriers, including the brain-, air-, and gut-blood barrier (BBB, ABB, and GBB, resp.) resulting in an uncontrolled “flooding” of tissues and microbial invasion. Physiologically, highly effective paracellular barriers are guaranteed by tight junctions, formed by various integral membrane molecules, such as occludin, claudin, and junctional adhesion molecule-1 (JAM-1/JAM-A/CD321/F11R) [3, 4]. In addition to the sealing of cell-cell contacts of endothelial and epithelial cells, JAM-1 is expressed on circulating cells, including leukocytes regulating transendothelial migration [5], or on hematopoietic stem cells facilitating long-term repopulation and reendothelialization of injured vascular walls [6, 7]. On a molecular level, JAM-1 can dimerize and bind homophilically via its extracellular domain, whereas its intracellular domain can interact with submembranous structures and signaling proteins [8]. JAM-1 is also present on platelets, where it can dimerize or cross-link with Fc*γ*RII, resulting in enhanced platelet adhesiveness and hyperaggregability [9]. Other reports suggest that JAM-1 in resting platelets inhibits premature platelet activation [10]. During inflammatory conditions, including murine autoimmune encephalomyelitis, a significant reduction in tight junction molecules, particularly JAM-1, was found to be associated with BBB dysfunction [11]. Similarly, ABB dysfunction, as modelled by lipopolysaccharide- (LPS-) induced acute lung injury, revealed partially reduced JAM-1 and claudin expression within the lungs [12]. In accordance with this, JAM-1-deficient mice exhibited classical signs of enhanced pulmonary permeability and susceptibility to a remote LPS challenge, suggesting JAM-1 as a key regulator of lung barrier function [13]. Under trauma conditions, ischemia and reperfusion (I/R) injury is a central pathophysiological driver of the inflammatory response. After induction of experimental liver I/R injury, JAM-1 deficiency did not alter hepatocellular necrosis but increased apoptotic rates and attenuated neutrophil infiltration [14]. Release of hemoglobin from extravasated erythrocytes (e.g., after brain injury), mimicked by stereotactic hemoglobin injection into the rat brain, led to a BBB breakdown with significantly reduced JAM-1 expression levels and enhanced nitric oxide production [15]. Furthermore, during chronic inflammatory diseases, including atherosclerosis, JAM-1 can be released from tight junctions into the circulation [16].

However, no data is currently available on a possible JAM-1 release in the context of severe tissue trauma. Therefore, we hypothesized that JAM-1 may be detected in the circulation after polytrauma, reflecting the degree of tissue injury. For the first time, we describe the detection of circulating JAM-1 in a murine polytrauma model and in patients after polytrauma which appears to be injury-pattern- and time-dependent and may therefore represent a useful future clinical tool to assess posttraumatic barrier dysfunction.

## 2. Materials and Methods

### 2.1. Experimental Murine Polytrauma

The study protocol for the experimental murine polytrauma was approved by the University Animal Care Committee and the Federal Authorities for animal research, Tuebingen, Germany (number 1016), and all experiments were performed in adherence to the National Institutes of Health Guidelines for the use of laboratory animals. C57BL/6 mice aged 8-9 weeks (Jackson Laboratories, Bar Harbour, USA) with a mean body weight of 25 g (±2.5 g) were used. Mice were anesthetized using a mixture of 2.5% sevoflurane (Sevorane, Abbott, Wiesbaden, Germany) and 97.5% oxygen and were exposed to blunt chest trauma or sham procedure. Following trauma, anesthesia was continued intraperitoneally using Ketavet (Pfizer Pharma, Karlsruhe, Germany) and Xylazine (Bayer Health Care, Monheim, Germany). Mice were randomly divided into the following treatment groups with *n* = 6–12 animals in each: control (sham), bilateral blunt chest trauma (TxT), closed traumatic brain injury (TBI), proximal femoral fracture including contralateral soft tissue injury (Fx + STI) or a combination of the traumata: TxT + TBI, TxT + Fx + STI, and TBI + Fx + STI, or polytrauma (PT = TxT + TBI + Fx + STI) as previously described [17]. Sham animals underwent identical procedures, but without trauma application. Mice were maintained anesthetized until sacrifice. Blood was withdrawn 2 h after trauma by cardiac puncture; plasma was obtained by centrifugation and stored at −80°C until further analysis. Lungs were either used for bronchoalveolar lavage (BAL) or homogenized for ELISA analysis.

### 2.2. Bronchoalveolar Lavage (BAL) Protein

BAL was collected (*n* = 6 mice per group) to assess the integrity of the alveolocapillary barrier. After sacrifice, the mouse trachea was exposed and cannulated, and the lung was flushed three times using 500 *μ*L ice-cold phosphate-buffered saline (Gibco, Eggenstein, Germany), including 10 *μ*L 1 : 1,000 broad-spectrum protease inhibitor (Sigma-Aldrich, St. Louis, MO). Subsequently, the BAL fluids were centrifuged at 380 ×g for 10 min at 4°C, and the supernatant was stored at −80°C until analysis. Protein concentrations in BAL fluids were determined using a BCA Protein Assay Kit (Pierce, Rockford, IL, USA) as recommended by the manufacturer using a microplate reader (Tecan GmbH, Grödig, Austria).

### 2.3. Measurement of JAM-1

Concentrations of tight junction protein JAM-1 were determined using a sandwich-enzyme-linked immunosorbent assay technique according to the manufacturer's recommendations. JAM-1 was determined in human (human JAM-1 ELISA, Cloud-Clone Corp., Houston, TX, USA) and murine plasma (mouse JAM-1 DuoSet ELISA, R&D, Minneapolis, MN, USA) as well as in lung tissue homogenates of mice after experimental trauma or sham procedure.

### 2.4. Polytrauma Patients

We conducted a prospective clinical study including patients with multiple trauma (Injury Severity Score (ISS) ≥ 18) who were admitted to the University Hospital Ulm between 2010 and 2011. The study was approved by the Independent Local Ethics Committee of the University of Ulm, number 69/08. Ten healthy volunteers served as a control group. Written informed consent was obtained from all the patients and volunteers. Exclusion criteria were age <18 years, pregnancy, infection with the human immunodeficiency virus, cardiogenic shock as the primary underlying disease, underlying hematologic disease, cytotoxic therapy given within the previous 6 months, and the presence of a rapidly progressive underlying disease anticipating mortality within the following 24 hours. In the trauma cohort, 8 multiple-injured patients (6 men, 2 women) with a mean age of 51 years (±24 years, standard deviation (SD)) and a mean ISS of 30 points (±4 points, SD) were enrolled to determine the JAM-1 plasma concentration. Blood samples were drawn on admission at the emergency room as well as 4, 12, 24, 48, 120, and 240 hours afterwards, and plasma aliquots were stored at −80°C until analysis.

### 2.5. Statistical Analysis

All results are given as the mean ± standard deviation (SD). Normal distribution of data was verified using the Kolmogorov-Smirnov test. Data sets were then analyzed using one-way analysis of variance (ANOVA) followed by the Dunnett method as a post hoc test for multiple comparisons. In case of nonparametric distribution, Kruskal-Wallis one-way analysis of variance on ranks followed by Dunn's method was performed. The Pearson method was used to analyze correlations between values. The results were considered as significant with *P* ≤ 0.05.

## 3. Results

### 3.1. Detection of JAM-1 in Plasma after Experimental Polytrauma: Association with BAL Protein Leakage as Air-Blood-Barrier (ABB) Dysfunction Marker

**Figure 1:**
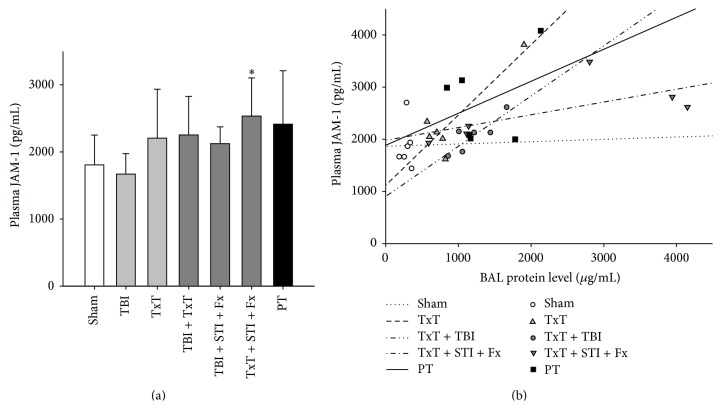
(a) JAM-1 levels in murine plasma 2 h after experimental blunt chest trauma (TxT), closed traumatic brain injury (TBI), femur fracture including contralateral soft tissue injury (STI + Fx), and combination of injuries (PT) (*n* = 6–9 per group) compared to sham procedure (*n* = 12). Results are presented as means ± SD; Kruskal-Wallis ANOVA on ranks followed by Dunn's method; ^*∗*^ *P* ≤ 0.05 versus sham. (b) Correlation analysis was performed between murine JAM-1 plasma levels and protein concentration in bronchoalveolar lavage (BAL) fluids to assess the impairment of the barrier function 2 h after blunt chest trauma (TxT), TxT in combination with traumatic brain injury (TxT + TBI), TxT combined with femur fracture and contralateral soft tissue injury (TxT + STI + Fx), polytrauma (PT), and sham procedure. Pearson correlation coefficient (*r*) of plasma JAM-1 versus BAL protein: *r* = 0.01 with *P* = 0.99 (sham); *r* = 0.89 with *P* = 0.02 (TxT); *r* = 0.86 with *P* = 0.03 (TxT + TBI); *r* = 0.67 with *P* = 0.10 (TxT + STI + Fx); and *r* = 0.36 with *P* = 0.48 (PT). Symbols show values for individual animals; lines represent linear regression for the indicated groups.

To determine whether JAM-1 as a key barrier molecule can be detected in the circulation early after tissue trauma, the JAM-1 plasma concentration was investigated in the mice 2 h after different standardized trauma insults. Indeed, there was a slight increase of circulating JAM-1 after thorax trauma alone (TxT) which was markedly increased in combination with an additional soft tissue and fracture trauma (TxT + STI + Fx) or polytrauma (Figure 1(a)). The amount of BAL protein leakage as indicator of an ABB dysfunction was significantly associated with JAM-1 plasma levels among all the animals (Pearson correlation coefficient *r* = 0.57; *P* < 0.01, data not shown). Herein, the blunt chest trauma insult (TxT) alone (*r* = 0.89; *P* = 0.02) or in combination with traumatic brain injury (TxT + TBI) (*r* = 0.86; *P* = 0.03) displayed a significant correlation with JAM-1 release (Figure 1(b)), suggesting that a trauma-induced dysfunction of the alveolocapillary barrier might to some extent be monitored from the plasma JAM-1.

### 3.2. Local Reduction of JAM-1 in Lung Tissue after Experimental Trauma

**Figure 2:**
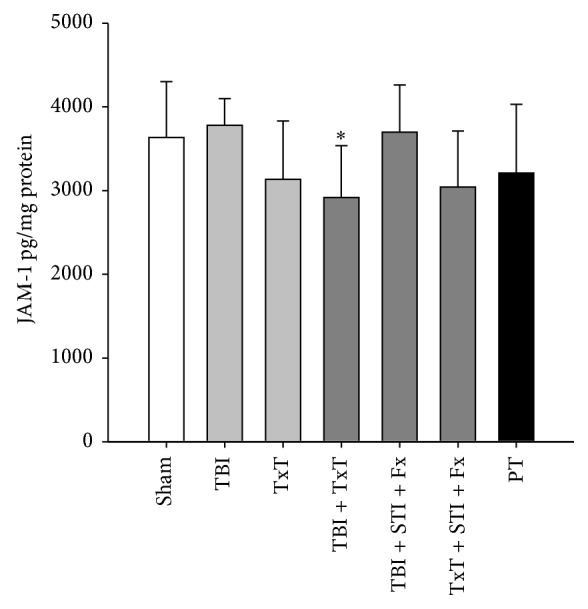
JAM-1 concentration of murine lung tissue homogenates per mg protein 2 h after experimental blunt chest trauma (TxT), closed traumatic brain injury (TBI), femur fracture including contralateral soft tissue injury (STI + Fx), and a combination of injuries (PT). Results are presented as means ± SD with *n* = 7-8 per group; one-way ANOVA/Dunnett's test; ^*∗*^ *P* ≤ 0.05 versus sham.

Because JAM-1 was increased in plasma mainly after experimental traumata that affected the lung (TxT; TxT + STI + Fx; and PT), we investigated the JAM-1 level in lung tissue homogenates. A slight decrease of JAM-1 in lung homogenates was found 2 h after blunt chest trauma alone (TxT), after an additional fracture and soft tissue injury (TxT + Fx + STI), and after polytrauma compared to sham animals (Figure 2). The combination of thorax trauma with traumatic brain injury (TxT + TBI) led to a significant decrease of JAM-1 in lung homogenates, suggesting that tissue injury leads to signs of ABB disruption as assessed by reduced JAM-1 tight junction protein in traumatized lungs.

### 3.3. Systemic Detection of JAM-1 in Human Polytrauma

**Figure 3:**
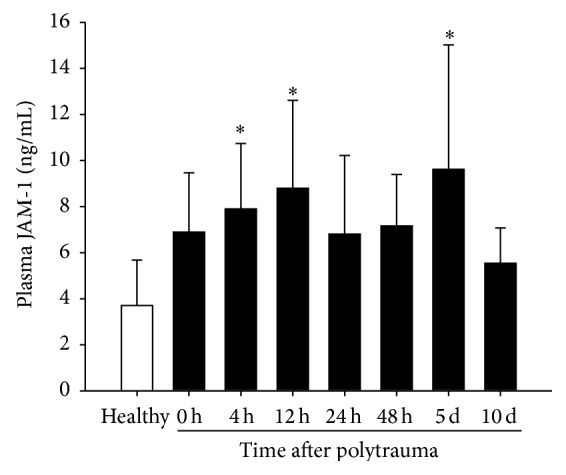
JAM-1 levels in plasma of polytrauma patients at the indicated time points after trauma. Data are presented as means ± SD with *n* = 6–10 per group; one-way ANOVA/Dunnett's test; ^*∗*^ *P* ≤ 0.05 versus healthy volunteers.

To investigate whether early trauma-induced barrier dysfunction can be monitored* in realiter*, JAM-1 plasma concentrations were determined in patients after severe multiple injuries and compared to healthy controls. There was a significant increase in plasma JAM-1 as early as 4 h after the trauma insult following a biphasic posttraumatic time course with maximal JAM-1 levels at 12 h and 120 h after injury (Figure 3). At 10 d after hospital admission, the JAM-1 concentrations of polytrauma patients had almost returned to control levels.

### 3.4. Association of Plasma JAM-1 with the Clinical Course Assessed by APACHE II and SOFA Scoring

**Figure 4:**
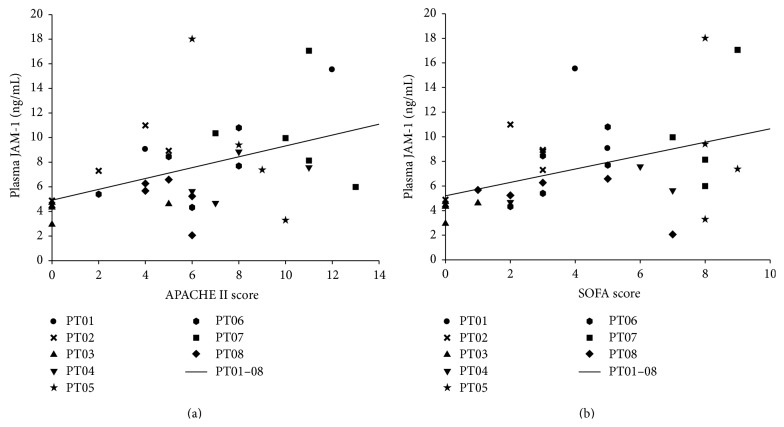
Correlation analysis between JAM-1 plasma levels of polytrauma patients (up to 10 d after trauma) and (a) the APACHE II score (Pearson correlation coefficient *r* = 0.50, *P* = 0.01) and (b) SOFA score (Pearson correlation coefficient *r* = 0.37, *P* < 0.04).

To determine whether JAM-1 release is associated with the severity of disease and degree of organ dysfunction, correlation analyses between JAM-1 plasma levels of polytrauma patients and established corresponding clinical scores were performed. JAM-1 plasma levels of the patients determined at 0, 24, 48, 120, and 240 h after trauma positively correlated with the “acute physiology and chronic health evaluation II” (APACHE II) score [18] (Figure 4(a)) and with the “sequential organ failure assessment” (SOFA) score [19] (Figure 4(b)). These results indicate that JAM-1 plasma concentration might, to some extent, mirror the severity of organ damage and dysfunction by following a temporal pattern after severe trauma.

## 4. Discussion

The present study is to our knowledge the first analysis demonstrating a trauma-induced release of tight junction protein JAM-1 into the circulation. We found that plasma JAM-1 increased in response to experimental murine polytrauma and in human polytrauma. In mice, JAM-1 levels were positively associated with BAL protein levels as an early sign of alveolocapillary barrier dysfunction. Furthermore, in patients, plasma JAM-1 correlated with severity of the disease and organ dysfunction as assessed by the APACHE II and SOFA scores, respectively, suggesting that JAM-1 might represent a marker for early trauma-induced barrier dysfunction.

Severe tissue injury causes direct cell and organ injury with disruption of vessels and tissue barriers, leading to transit problems for air, blood, liquor, urine, and feces. Additionally, remote barrier failure with end-organ dysfunction may develop as a result of the systemic danger response after severe tissue trauma. In regard to trauma-induced ABB dysfunction, some clinical data are available. In patients with severe polytrauma (ISS > 40), sequential measurements of BAL fluids revealed signs of an increased alveolocapillary permeability within 6 h after trauma, although none of them suffered a severe lung contusion. The ABB dysfunction was significantly aggravated in those patients who later developed acute respiratory distress syndrome [20, 21]. In our murine trauma model, multiple trauma led to a significant increase of lung myeloperoxidase as a marker for airway inflammation, even in the group that received no chest trauma [17]. Regarding trauma-induced breakdown of the GBB, intestinal-epithelial-cell damage was found to correlate with both the abdominal injury severity (Abbreviated Injury Scale (AIS)) and the general injury severity (determined by ISS), indicative of direct and remote intestinal damage after severe tissue trauma [22]. In a porcine multiple firearm-injury model, multiple trauma caused remote gastrointestinal ischemia and GBB failure with detection of gut-derived endotoxin and diamine oxidase in the portal vein within 6 h even in the absence of a hemorrhagic shock [23].

In the regulation of endogenous blood-organ barrier function, intercellular junctions and particularly tight junctions play a central role [24]. Disruption of the tight-junction structure is a common feature of many evident or hidden inflammatory diseases [24–27], including sepsis [28], shock, and trauma [29, 30]. Several studies suggested that JAM-1 represents a key player in tight junction assembly and regular barrier function. In SK-CO15 intestinal cells, downregulation of JAM-1 resulted in increased epithelial permeability [31].* In vivo*, JAM-1-knockout mice displayed enhanced mucosal permeability as shown by increased dextran passage and decreased transepithelial resistance. Yeung et al. investigated the role of JAM-1 in BBB integrity under inflammatory conditions. In a rat model of cortical cold injury, dual labeling of JAM-1 and fibronectin revealed that only lesioned vessels with BBB breakdown displayed a loss of brain endothelial JAM-1 immune staining [32]. Reduced endothelial JAM-1 immunostaining was also demonstrated in active brain lesions of patients with multiple sclerosis [33]. In primary alveolar epithelial cells, depletion of JAM-1 resulted in increased epithelial permeability as indicated by decreased transepithelial resistance, decreased expression of scaffold tight junction protein zonula occludens 1, and disorder of the structural transmembrane protein claudin 18 [13].* In vivo*, LPS intraperitoneal injection led to increased susceptibility to pulmonary edema and the reduction or disorder of associated tight-junction proteins in JAM-1-knockout mice, suggesting that JAM-1 is essential for the regulation of tight junction interaction and lung-barrier function [13]. Accordingly, in the present polytrauma model, we found that lung contusion led to an early breakdown of the alveolar barrier as assessed by increased BAL protein, which was associated with a reduction of JAM-1 in lung homogenates and an increase of JAM-1 in the plasma of mice. In the polytrauma patient cohort, 7 out of 8 patients suffered from injuries to the thorax (mean AIS = 3), suggesting that severe organ damage might cause disruption of paracellular tight junctions and the subsequent release of barrier molecules, including JAM-1, into the circulation. In addition to mechanical barrier breakdown, inflammation is considered a pivotal trigger of JAM-1 release. Upon proinflammatory stimulation of human umbilical vein endothelial cells (HUVECs), JAM-1 was shed from the cell surface by a disintegrin and metalloproteinases 10 and 17 [34]. Additionally, JAM-1 shedding was increased in HUVECs 2 h after incubation with stimulated neutrophils. In mice, injection of IFN-*γ* and TNF-*α* (both factors are elevated after polytrauma in humans) induced a systemic inflammation, which was associated with increased levels of soluble JAM-1 in the circulation within 2.5 h. Although severe tissue trauma is a well-known progressor of the inflammatory response [2, 35, 36], trauma-induced shedding of JAM-1 is relatively unexplored. In the present mouse model, polytrauma led to a systemic increase of IL-6 [17] and JAM-1, indicating that trauma-induced inflammation might represent a trigger for tight junction release and subsequent barrier dysfunction. Accordingly, in patients, CRP as a clinical inflammatory monitoring marker was significantly elevated starting 12 h after polytrauma (data not shown) as a sign for trauma-induced systemic inflammation. However, whether CRP or IL-6 causally contribute to JAM-1 release after trauma remains to be clarified. Furthermore, JAM-1 plasma levels were associated with disease severity (APACHE II) and organ dysfunction (SOFA), indicating a relationship between the breakdown of paracellular junctions and organ damage.

As a limitation of the study, the present multiple-injured patient cohort was too small for powerful subgroup analysis to satisfactorily answer further clinical questions, including a theoretical association of JAM-1 concentrations with distinct injury patterns and the development of subsequent complications (e.g., hemorrhagic shock, disseminated intravascular coagulopathy, and septic shock). It has to be clarified whether JAM-1 is shed from the endothelium, leukocytes, platelets, or hematopoietic stem cells. Furthermore, we did not determine whether the released JAM-1 protein represents the full-length molecule capable of redimerizing homophilically or whether we merely detected a cleavage product. Consideration should be given to the fact that JAM-1 shedding might additionally serve as a self-defense mechanism. In this regard, Koenen et al. demonstrated that the shed cleavage form of JAM-1 was able to reduce transendothelial migration of neutrophils* in vitro* and* in vivo*, suggesting that soluble JAM-1 might regulate vascular permeability [34]. Regarding physiological consequences, increased circulating plasma concentrations of shed JAM-1 may reduce migration of leukocytes [34], repopulation of hematopoietic stem cells [7], reendothelialization of injured vessel walls [6], and adhesion of platelets [10]. Thus, elevated JAM-1 plasma levels may stage-dependently reflect beneficial or harmful functional effects during the course of traumatized patients.

For the clinical application, a bedside barrier molecule monitoring would be desirable especially since JAM-1 seems to be different from other markers used in the clinic as it is not considered an inflammatory molecule monitoring the patient's inflammatory state; given its function as a central barrier molecule, blood levels might therefore provide information on the state of the patient's blood-organ barriers and predict their breakdown even before appearance of clinically evident signs of leakage syndrome, such as edema.

The current finding that the barrier molecule JAM-1 is present in plasma early after experimental and clinical polytrauma may help to detect and possibly monitor early barrier and organ dysfunction and, furthermore, the inflammatory response, particularly because it remains unclear whether the barrier dysfunction represents the cause or the consequence of the systemic inflammatory response. The identification of barrier dysfunction markers may aid in the recognition of patients at risk of posttraumatic leakage syndrome and, thereby, improve the clinical management of severely injured patients.
